# The effect of multimedia-based education on nurses’ clinical capability in radiation protection in operating rooms: a randomized controlled study

**DOI:** 10.1186/s12912-025-04108-3

**Published:** 2025-12-15

**Authors:** Mehrvash Hemati, Sina Ghanbarzadeh, Zinat Mohebbi

**Affiliations:** 1 Community Based Psychiatric Care Research Center, School of Nursing and Midwifery, Shiraz University of Medical Sciences, Shiraz, Iran; 2 Student Research Committee, School of Medicine, Shiraz University of Medical Sciences, Shiraz, Iran; 3 Department of Nursing, School of Nursing and Midwifery, Shiraz University of Medical Sciences, Shiraz, Iran

**Keywords:** Multimedia-based education, Radiation protection, Operating room nurses, Clinical capability

## Abstract

**Background:**

Radiation exposure is a significant occupational hazard for operating room nurses, yet their knowledge and practice of radiation protection are often inadequate. Enhancing nurses’ clinical capability in this area is crucial for safety and quality care. Multimedia-based education offers an effective, flexible approach to improve learning and adherence to safety protocols. This study aimed to determine the effect of multimedia-based education on nurses’ clinical capability in radiation protection in operating rooms.

**Methods:**

This randomized controlled trial was conducted on 100 operating room nurses from two hospitals affiliated with Shiraz University of Medical Sciences between June and December 2024. Participants were randomly assigned to the intervention (*n* = 50) and control (*n* = 50) groups. The intervention group received a two-week multimedia-based educational program on radiation protection via WhatsApp, while the control group received routine hospital training. Primary outcomes included clinical capability assessed through a validated questionnaire covering knowledge, attitude, performance, and personal commitment, measured at baseline, one month, and two months post-intervention.

**Results:**

After the intervention, the intervention group showed significant improvements in all dimensions of clinical capability, including knowledge, attitude, performance, and personal commitment, compared to the control group. Clinical capability scores at one and two months post-intervention were significantly higher in the intervention group (all *p* < 0.001). The effect sizes ranged from 0.79 to 2.73, indicating large effects. ANCOVA analysis revealed a significant interaction between group and time (F = 32.8, *p* < 0.001), confirming that the improvements over time were greater in the intervention group than the control group after adjusting for baseline values.

**Conclusion:**

Therefore, we recommend implementing multimedia-based education to enhance clinical capability in radiation protection among operating room nurses.

**Trail registration number:**

Registration number IRCT20211129053216N1 on March 19, 2024 (https://www.irct.ir).

## Background

Every day in operating rooms around the world, nurses stand just a few steps away from invisible yet potentially harmful radiation [1]. Radiographic and fluoroscopic examinations have become indispensable tools in modern surgery, enabling real-time guidance that enhances precision and improves patient safety [2]. Yet, while these technologies benefit patients, they simultaneously expose operating room personnel to ionizing radiation—a silent occupational hazard with well-documented risks [3]. Long-term exposure has been associated with cataracts, thyroid disorders, genetic mutations, hematologic abnormalities, and an increased incidence of cancer [4–7], with a study showing a four-fold higher risk compared to personnel without radiation exposure [6].

Protecting operating room personnel from occupational radiation is essential for both individual health and safe patient care [8]. However, evidence indicates that nurses’ knowledge and adherence to radiation safety protocols remain suboptimal [3, 9]. Globally, evidence from Morocco, Thailand, and Egypt shows limited knowledge of radiation hazards, inconsistent use of protective equipment, and suboptimal adherence to safety protocols [3, 10, 11]. In Iran, recent findings confirm that despite moderate awareness of radiation safety principles, adherence to protective protocols remains inadequate among operating room nurses [12]. Contributing factors in Iranian operating rooms include high-paced surgical environments, limited opportunities for hands-on training, and insufficient coverage of radiation protection in academic curricula [12–15]. Therefore, strengthening nurses’ clinical capability in radiation protection is essential to bridge the gap between theoretical knowledge and safe practice in the operating room.

Clinical capability in radiation protection is the integrated ability of operating room nurses to combine knowledge, attitude, performance, and professional commitment in their daily work to minimize occupational exposure [16]. This competence reflects nurses’ ability to apply theoretical knowledge in practice and adhere to radiation safety standards [12]. Higher levels of clinical capability are associated with safer practices, improved risk management, and better professional performance, ultimately enhancing both staff and patient safety [17, 18]. Therefore, it is essential to implement targeted educational interventions that strengthen clinical capability among operating room nurses.

To address these challenges, several educational interventions have been investigated to improve radiation protection knowledge and practices among healthcare professionals. While these programs generally show positive effects, their findings are limited by methodological shortcomings. Many interventions targeted broader groups of healthcare workers rather than specifically operating room nurses, limiting the applicability of findings to this high-risk population. Some studies employed quasi-experimental designs, had small sample sizes, or were conducted in single centers, reducing the strength and generalizability of results. In addition, most studies included only a single follow-up assessment, making it difficult to determine the sustainability of observed effects. Furthermore, several interventions focused solely on measuring knowledge rather than actual protective behaviors, leaving uncertainty about their impact on real-world clinical practice. Therefore, there remains an urgent need for robust, targeted educational approaches that are specifically designed for operating room nurses, include multiple follow-ups, and assess both knowledge and practical adherence to radiation safety standards [19–22].

In this context, multimedia-based educational interventions represent a promising educational approach to improve healthcare professionals’ knowledge and skills in clinical practice [23]. These interventions deliver information in a structured and engaging format, potentially facilitating improved comprehension and application of relevant principles [24]. For instance, a study comparing multimedia-based training with traditional instructional booklets demonstrated significantly greater gains in nurses’ awareness following the multimedia intervention. Nevertheless, this study had several methodological limitations, including its quasi-experimental design, relatively small sample size, and a single short-term follow-up, which may restrict the generalizability of the findings [25].

Although previous educational interventions have shown benefits in improving nurses’ knowledge and practice regarding radiation protection, evidence on programs specifically targeting operating room nurses remains limited. Moreover, most studies have primarily focused on knowledge and practical skills, with limited attention to nurses’ attitudes and professional commitment. In addition, studies using flexible and accessible approaches, such as multimedia- or mobile-based interventions, are scarce. In prior studies, follow-up assessments were typically conducted only once, limiting insight into the sustainability of learning outcomes. To address these gaps, the present study evaluated a multimedia-based educational program delivered through accessible platforms, designed to enhance operating room nurses’ clinical capability across four dimensions—knowledge, attitude, performance, and professional commitment—assessed through two follow-up evaluations, aiming to strengthen overall clinical capability in radiation protection.

## Methods

### Design and participants

This study is a two-group clinical trial with a pre-/post-test design conducted on 100 operating room nurses working in Rajaei and Chamran hospitals, affiliated with Shiraz University of Medical Sciences. These hospitals were selected because they have the highest number of X-ray procedures performed in the operating rooms. The study was conducted from the beginning of June to the end of December 2024. Based on a previous single-group pre–post study by Dauer et al. [17], the mean (SD) knowledge scores regarding radiation protection among nurses were 59 (13) before the intervention and 71 (20) after the intervention. Therefore, the sample size was calculated based on a paired-sample t-test using G*Power version 3.1. With a significance level (α) of 0.05 and a power (1 − β) of 90%, the minimum required sample size was estimated to be 44 participants. To account for an anticipated attrition rate of 5%, the final sample size was increased to 50 participants per group in this two-group randomized controlled trial. Based on official staffing data from the hospitals’ nursing departments, Rajaei Hospital employs 75 operating room nurses and Chamran Hospital 50. Accordingly, 60 participants were proportionally recruited from Rajaei Hospital and 40 from Chamran Hospital using stratified random sampling to ensure proportional representation.

Inclusion criteria were operating room nurses involved in surgeries requiring radiological interventions, with no history of genetic cancer or malignancies, no history of radiotherapy or diagnostic scans with radionuclides, no prior exposure to radioactive materials in previous jobs (such as benzene, arsenic, lead, or mercury), no family history of blood diseases, at least one year of experience in surgeries requiring radiological interventions, with access to WhatsApp messaging platform and willingness to participate in the study. Exclusion criteria included concurrent participation in any other radiation protection training program during the study period, unwillingness to continue participation in the study, and failure to complete the questionnaires.

**Fig. 1:**
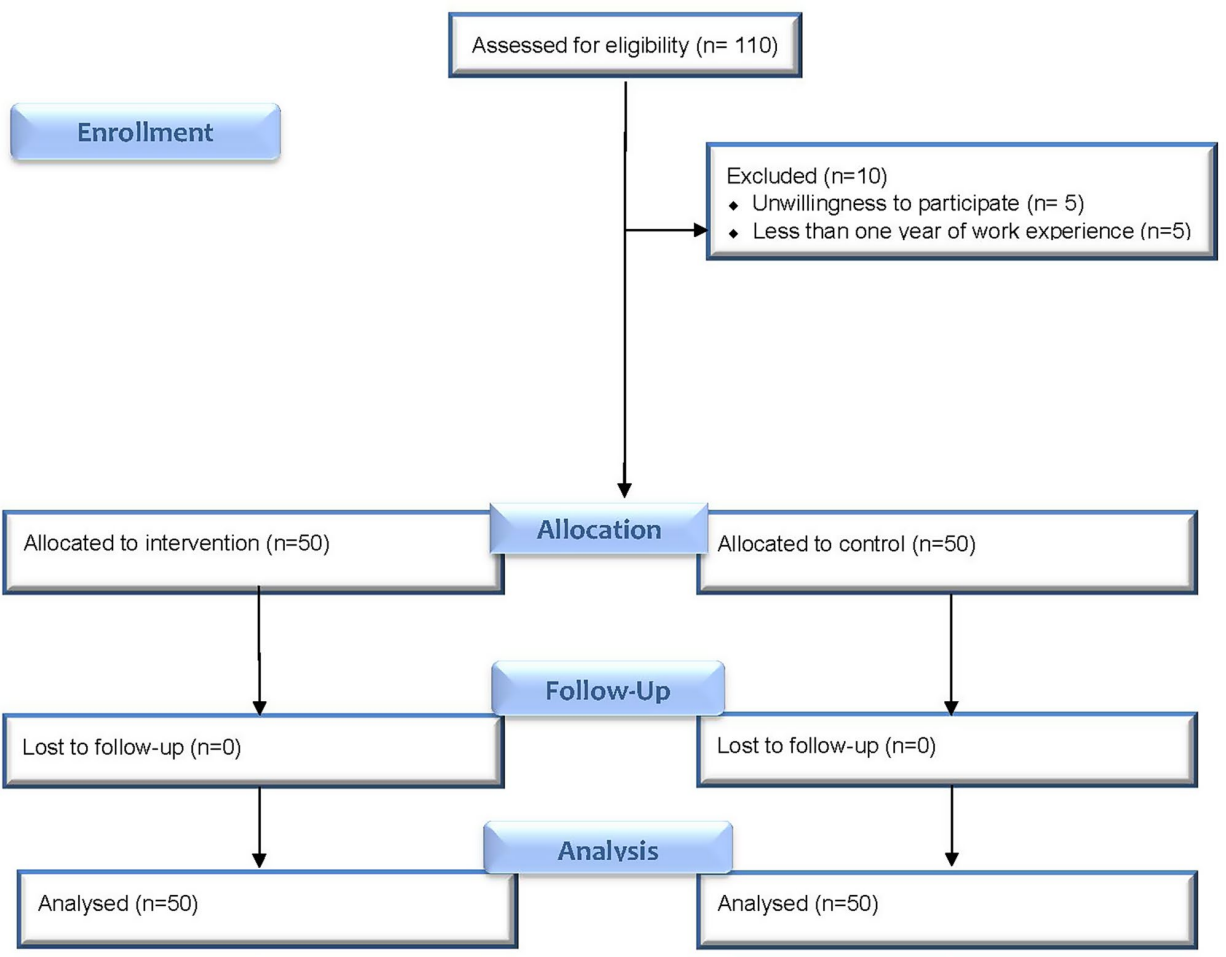
CONSORT flow diagram of participant enrollment, allocation, and analysis in the randomized controlled trial

Eligibility was determined according to predefined inclusion and exclusion criteria. Participants were randomly assigned in a 1:1 ratio to either the intervention group (*n* = 50) or the control group (*n* = 50) using block randomization with a block size of 4, generated by Random Allocation Software (version 2.0). The allocation sequence was concealed in opaque, sequentially numbered envelopes, which were opened by a research assistant blinded to group assignments upon each participant’s enrollment. To minimize contamination bias, the control group was recruited first and completed both pre- and post-test assessments before the enrollment of the intervention group started. This approach helped prevent information crossover between the groups. Furthermore, all outcome data were collected by a separate research assistant who was blinded to group allocation, ensuring unbiased data collection. The CONSORT diagram illustrating the selection process and exclusions is shown in Fig. 1.

#### Intervention

This multimedia educational intervention was designed to enhance operating room nurses’ clinical capability in radiation protection. The program delivered evidence-based training materials over two weeks through the WhatsApp messaging platform. The WhatsApp messaging platform was chosen due to its wide accessibility among Iranian nurses, allowing nurses to access educational materials at convenient times, and its ability to support interactive learning, which is particularly suitable for busy operating room staff. The intervention comprised a structured curriculum, including five PowerPoint presentations, three brochures, and two posters, all tailored to address critical aspects of radiation safety in the operating room. The educational content was developed based on authoritative sources, including guidelines from the International Commission on Radiological Protection (ICRP), the World Health Organization (WHO), the International Atomic Energy Agency (IAEA), and national radiation safety standards.

In the first week, participants received three PowerPoint presentations covering fundamental principles of radiation protection in the operating room, best practices for staff protection against C-arm radiation exposure, and patient protection with a focus on pregnant women and pediatric patients. Alongside these presentations, participants also received a brochure outlining radiation protection recommendations for women, pregnant patients, and radiology, and a poster highlighting essential radiation protection strategies for patients and staff in diagnostic and interventional radiology.

During the second week, two additional PowerPoint presentations were delivered, focusing on an introduction to protective equipment used in the operating room, radiation incidents, and strategies for their management. Additionally, participants received two brochures detailing protective measures for the staff in fluoroscopy and interventional radiology environments and ten key radiation safety tips for the personnel working in fluoroscopy and radiography.

Each presentation provided both theoretical knowledge and practical guidelines to reinforce safe practices. The educational materials were designed to promote awareness and improve radiation safety behaviors in the operating room setting. To ensure adherence, participants were asked to confirm receipt and completion of each module. At the end of the intervention period, a follow-up session was conducted to address the participants’ questions and reinforce critical radiation protection practices. The control group continued to receive routine radiation protection education as provided by the hospital’s standard training programs during the study period. However, they did not receive the specialized multimedia-based educational intervention. To address ethical considerations, all educational materials used in the intervention group were made available to the control group after completion of the post-test assessments.

### Data collection

Data were collected using a demographic information questionnaire and the Clinical Capability Assessment Scale at three time points, namely before the intervention, one month after the intervention, and two months after the intervention. Both the intervention and control groups completed the questionnaires at all three time points.

#### The demographic information questionnaire

The demographic form included questions about age, gender, marital status, educational degree, field of work, career role, predominant work shift, employment status, years of experience in the operating room, years of experience as a radiation-exposed nurse, and the previous radiation protection training.

#### Clinical capability assessment scale

Mohebbi et al. (2021) developed this questionnaire and validated its psychometric properties. It included 32 items divided into four categories: knowledge (13 items), attitude (10 items), performance (5 items), and personal commitment (4 items). Responses were measured using a five-point Likert scale. The questionnaire was constructed based on an extensive literature review and input from experts. Its face and content validity were confirmed using the Content Validity Ratio (CVR) and Content Validity Index (CVI), leading to the removal of unsuitable items. Construct validity was assessed through exploratory factor analysis, which identified a four-factor structure accounting for 54.4% of the total variance. The reliability of the questionnaire and its subscales was confirmed, with Cronbach’s alpha coefficients ranging between 0.50 and 0.76 [16].

#### Data analysis

Data were analyzed using SPSS software, version 22.0 (IBM Corp., Armonk, NY, USA). The Shapiro–Wilk test was used to assess the normality of the data distribution. Categorical variables, including gender, marital status, educational degree, field of work, employment status, predominant work shift, previous radiation protection training, and career role, were compared between the intervention and control groups using the Chi-square and Fisher’s exact test. Continuous variables such as age, years of experience in the operating room, and years of experience working with radiation did not follow a normal distribution; therefore, between-group comparisons for these variables were performed using the Mann–Whitney U test.

For outcome variables, the subscales of knowledge, performance, and personal commitment were non-normally distributed and analyzed using non-parametric tests (Mann–Whitney U test for between-group comparisons and Friedman test for within-group comparisons). The attitude subscale showed a normal distribution; therefore, within-group comparisons across the three time points were conducted using repeated measures ANOVA, and between-group differences at each time point were analyzed using independent samples t-tests. To evaluate the interaction effect of time and group, analysis of covariance (ANCOVA) was employed. Cohen’s d effect size was calculated for between-group changes. The effect sizes of 0.8, 0.5, and 0.2 were considered large, medium, and weak, respectively [26]. *P* < 0.05 was considered statistically significant.

## Results

Among the 100 participants enrolled in the study, all completed the intervention and follow-up assessments. The mean (SD) age of the participants was 37.0 (1.1) years in the intervention group and 36.6 (1.0) years in the control group (P-value = 0.896). The average duration of operating room experience was 13.8 (1.2) years in the intervention group and 12.3 (1.0) years in the control group (P-value = 0.510). Moreover, the mean (SD) years of experience as a radiation-exposed nurse 10.0 (1.0) and 9.1 (0.8) in the intervention and control groups, respectively (P-value = 0.830). No statistically significant differences were found between the two groups regarding baseline demographic characteristics (Table 1).

**Table 1:** Baseline demographic and clinical characteristics of participants

Variables		Total (*n* = 100)	Intervention (*n* = 50)	Control (*n* = 50)	P-value
		n (%)	n (%)	n (%)	
Gender	Female	52 (52%)	24 (48%)	28 (56%)	0.423*
	Male	48 (48%)	26 (52%)	22 (44%)	
Marital status	Single	34 (34%)	19 (38%)	15 (30%)	0.398*
	Married	66 (66%)	31 (62%)	35 (70%)	
Career role	Nurse anesthetist	41(41%)	18 (36%)	23 (46%)	0.309*
	Surgical technologist	59 (59%)	32 (64%)	27 (54%)	
Field of work	Orthopedics	62 (62%)	34 (68)	28 (56%)	0.216*
	Neurology	38 (38%)	16 (32%)	22 (44%)	
Predominant work shift	Morning	20 (20%)	12 (24%)	8 (16%)	0.317*
	Rotating shift	80 (80%)	38 (76%)	42 (84%)	
Employment status	Permanent	86 (86%)	42 (84%)	44 (88%)	0.564*
	Contractual/Mandatory Service	14 (14%)	8 (16%)	6 (12%)	
Previous radiation protection training	Yes	75 (75%)	36 (72%)	39 (78%)	0.488*
	No	25 (25%)	14 (28%)	11 (22%)	
Educational degree	Bachelor’s degree	96 (96%)	46 (92%)	50 (100%)	0.488**
	Master’s degree	4 (4%)	4 (8%)	0 (0%)	

* The p-values were obtained using the Pearson Chi-square test

** The p-value was obtained using Fisher’s exact test

The results showed no significant differences between the two groups in any dimension of clinical capability before the intervention (all P-values > 0.05). The 95% confidence intervals (CI) for baseline effect sizes included zero or negative values, reflecting no initial differences between groups. However, at both the one-month and two-month follow-ups, the intervention group demonstrated significantly higher scores in knowledge, attitude, performance, and personal commitment compared to baseline (all P-values < 0.001). Moreover, post-intervention scores in all dimensions were significantly higher in the intervention group than in the control group (all P-values < 0.001). The effect sizes for between-group differences in clinical capability scores ranged from 0.789 to 2.730, indicating large effect sizes (Table 2).

**Table 2:** Clinical capability scores of intervention and control groups at baseline, 1- and 2-month after

Clinical capability assessment scale	Evaluation time	Group		*P*-value (between-groups)	Cohen’s d effect size	95% CI for Cohen’s d effect size
		Intervention *n* = 50	Control *n* = 50			
		Mean ± SD Median (IQR)	Mean ± SD Median (IQR)			
Knowledge	**Baseline**	41.2 ± 1.3 42.0 (12.5)	41.7 ± 1.3 45.5 (13.0)	0.717*	0.056	[-0.448, 0.336]
	**1-month after**	60.3 ± 0.5 61.0 (7.2)	41.2 ± 1.2 43.0 (12.2)	0.001*	2.730	[2.179, 3.273]
	**2-month after**	58.1 ± 0.7 58.0 ( 8.2)	41.3 ± 1.2 43.5 (11.5)	0.001*	2.335	[1.822, 2.842]
**P-value (within-groups)**		0.001***	0.168***			
Attitude	**Baseline**	33.7 ± 1.0 33.0 (11.2)	33.3 ± 0.8 34.0 (8.2)	0.209**	0.065	[-0.327, 0.457]
	**1-month after**	41.6 ± 0.6 42.0 (7.0)	32.7 ± 0.8 33.0 (7.2)	0.001**	1.677	[1.217, 2.131]
	**2-month after**	39.0 ± 0.7 38.5 (8.0)	32.8 ± 0.8 33.5 (8.2)	0.001**	1.126	[0.701, 1.546]
**P-value (within-groups)**		0.001****	0.939****			
Performance	**Baseline**	19.3 ± 0.7 21.0 (8.5)	18.3 ± 0.7 19.0 (6.5)	0.225*	0.185	[-0.209, 0.577]
	**1-month after**	23.4 ± 0.3 24.0 (3.0)	18.1 ± 0.6 19.0 (6.0)	0.001*	1.497	[1.050, 1.938]
	**2-month after**	22.5 ± 0.4 23.5 (5.0)	18.2 ± 0.6 18.0 (6.2)	0.001*	1.175	[0.747, 1.597]
**P-value (within-groups)**		0.001***	0.522***			
Personal commitment	**Baseline**	14.8 ± 0.4 15.0 (4.0)	14.4 ± 0.4 14.5 (4.0)	0.384*	0.141	[-0.251, 0.534]
	**1-month after**	16.8 ± 0.3 17.0 (2.2)	13.9 ± 0.4 14.0 (4)	0.001*	1.140	[0.715, 1.561]
	**2-month after**	16.1 ± 0.3 16.0 (2.0)	14.1 ± 0.4 14.0 (3.2)	0.001*	0.789	[0.380, 1.194]
**P-value (within-groups)**		0.001***	0.234***			

* The p-values were obtained using the Mann–Whitney U test

** The p-values were obtained using the independent samples t-test

*** The p-values were obtained using the Friedman test

**** The p-values were obtained using repeated measures ANOVA

The ANCOVA revealed no significant main effects for group (F(1, 294) = 4.343, *p* = 0.173, η² = 0.015) or time (F(2, 294) = 0.810, *p* = 0.552, η² = 0.448) on clinical capability scores. However, a significant group × time interaction was found (F(2, 294) = 32.845, *p* < 0.001, η² = 0.183). This indicates that the change in clinical capability scores over the assessment periods (before, 1 month after, and 2 months after the intervention) was dependent on the group. Specifically, the intervention group demonstrated greater improvement over time compared to the control group. Therefore, the significant change in clinical capability is attributable to the combined effect of receiving the intervention and the passage of time (Table 3).

**Table 3:** ANCOVA results for effects of group, time, and group × time interaction on clinical capability

Effect	Type III sum of squares	df	Mean square	F	*P*-value	ηp²
Group	36940.8	1,294	36940.8	4.343	0.173	0.685
Time	13779.3	2,294	6889.6	0.810	0.552	0.448
Group × Time	17010.6	2,294	8505.3	32.845	0.001	0.183

**Fig. 2:**
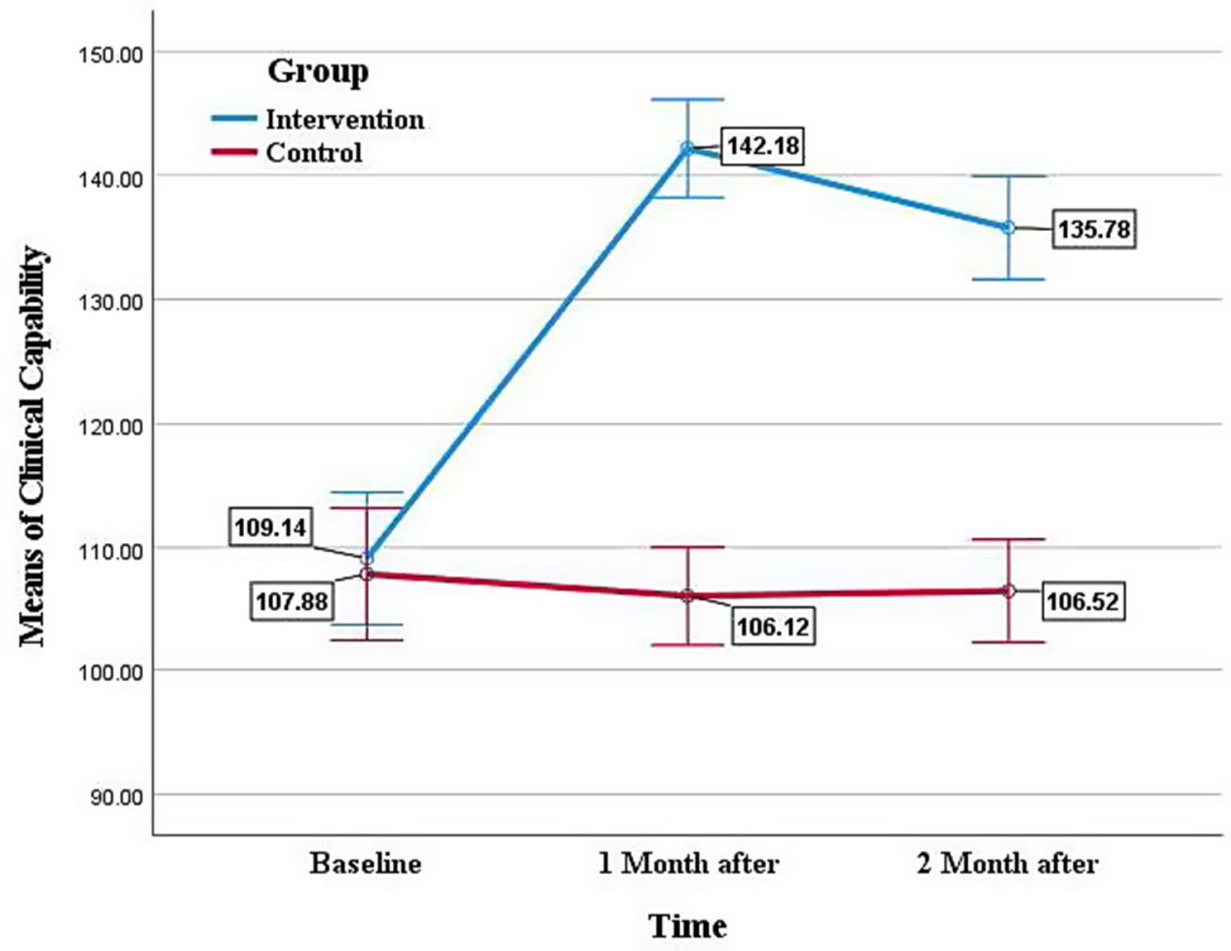
Adjusted mean ± SE of nurses’ clinical capability at baseline, 1 month, and 2 months after intervention (intervention: blue, control: red)

Figure 2 demonstrates the adjusted mean ± SE of nurses’ clinical capability at baseline, 1 month, and 2 months after the intervention.

## Discussion

The operating room environment is characterized by high workload and time constraints, which often limit nurses’ opportunities to participate in traditional, in-person training sessions [27]. Multimedia-based education allows nurses to access training materials at any time, regardless of their shift schedule or workload, overcoming common logistical barriers and ensuring consistent learning opportunities even in high-demand clinical settings [28, 29].

This randomized clinical trial was designed to evaluate the effectiveness of a multimedia-based educational intervention on operating room nurses’ clinical capability in radiation protection. At the end of the intervention, the intervention group showed significant improvements across all subscales—knowledge, attitude, performance, and personal commitment—compared to the control group. These improvements were sustained at both the one-month and two-month follow-ups, with only a slight decline at the second follow-up. In contrast, the control group showed no statistically significant changes across the three time points. The ANCOVA results indicated no statistically significant main effects of group or time alone on clinical capability scores; however, the group × time interaction was statistically significant, indicating that the observed improvements were specifically attributable to the multimedia-based intervention. This indicates that while neither group membership nor time alone significantly influenced clinical capability, the combined effect of being in the intervention group over time led to meaningful improvements. The significant post-test differences and large effect sizes further support the potential clinical relevance and utility of this educational approach. These findings suggest that multimedia-based education may meaningfully enhance the clinical capability of operating room nurses in radiation protection.

These findings are also consistent with previous literature demonstrating that educational interventions can effectively enhance nurses’ knowledge of radiation protection. For instance, Osanai et al. (2025) found that video-based educational materials significantly improved knowledge scores among nursing students and practicing nurses [19]. Likewise, Wahab and Younis (2025) reported that lecture-based education led to higher knowledge levels among non-operating room nurses [30]. Although both studies focused solely on knowledge outcomes and involved different nursing populations, our multimedia-based intervention extended these approaches by delivering flexible, WhatsApp-based learning accessible across work shifts and tailored specifically for operating room nurses. Collectively, this evidence highlights the pivotal role of educational interventions in strengthening nurses’ knowledge of radiation safety.

A quasi-experimental study by Charmchi et al. (2025) specifically evaluated multimedia-based training to improve awareness of radiation protection principles among operating room personnel. Their results showed that while both multimedia and booklet-based interventions increased awareness compared to a control group, the multimedia-based method produced notably superior outcomes. However, the study involved a relatively small sample size, employed a quasi-experimental design, and included only a single follow-up assessment [25]. In contrast, the present study implemented a two-week intervention with two follow-up evaluations, which may have contributed to more sustained and reliable improvements not only in knowledge but also in performance, attitude, and personal commitment. These findings reinforce the potential value of multimedia-based interventions to enhance not only knowledge but also performance, attitude, and personal commitment in radiation protection, particularly within the operating room setting.

These findings are further supported by evidence indicating that technology-based education can effectively improve nurses’ performance in radiation protection. For instance, Hasanian et al. (2020) demonstrated that virtual learning delivered through a social network platform significantly enhanced practical adherence to X-ray protection principles among hospital nurses [22]. Similarly, Khamis et al. (2025) showed that immersive virtual reality training produced substantial and sustained reductions in radiation exposure among interventional radiology nurses [20]. While these studies affirm the potential of digital and simulation-based learning to promote safer radiation practices, our multimedia-based intervention offered a more accessible, low-cost, and context-specific alternative by integrating flexible, WhatsApp-delivered modules tailored for operating room nurses. Collectively, this body of evidence highlights the potential role of technology-enhanced education in translating knowledge into safer professional behaviors in radiation environments.

On the other hand, not all studies have reported consistently positive results for educational interventions in radiation protection. For example, a quasi-experimental study by Kim and Kang (2025) examined a one-week web-based program for operating room nurses [21]. While the intervention significantly improved attitudes and behaviors, it had no clear effect on knowledge acquisition compared to a control group. The limited duration and intensity of the intervention, along with its quasi-experimental design, may have contributed to this lack of effect on knowledge. This underscores that although educational programs generally enhance nurses’ capabilities, factors such as the length and intensity of the intervention, as well as the study design, can influence their effectiveness on different learning outcomes [21].

In the present study, educational content was delivered via WhatsApp, a widely accessible and user-friendly platform that enables flexible, asynchronous learning [31]. The use of this platform may be particularly advantageous for operating room nurses, whose variable schedules and clinical demands often restrict participation in traditional face-to-face training [32]. This delivery method not only facilitated continuous engagement but also supported interaction and feedback during the intervention period [33]. To sustain the positive effects observed in this study beyond the two-month follow-up, future programs could incorporate periodic refresher modules, interactive reminders, or blended approaches that combine multimedia-based materials with brief in-person or virtual reinforcement sessions. Implementing such strategies in future training programs may contribute to sustained knowledge and safer professional practices.

### Strengths and limitations

This study has several notable strengths. First, it is among the few randomized controlled trials to evaluate the effect of multimedia-based education on the comprehensive clinical capability of operating room nurses in the field of radiation protection. Unlike many previous studies that focused solely on knowledge or attitude, this study assessed multiple dimensions including knowledge, attitude, performance, and personal commitment, providing a more holistic evaluation of educational outcomes. Second, the use of two follow-up assessments allowed for monitoring both immediate and short-term retention of learning, offering insights into the sustainability of the intervention’s impact. Third, the intervention was designed to be feasible and scalable, utilizing a widely accessible platform (WhatsApp), which enhances its applicability in real-world clinical settings, especially in high-demand environments such as operating rooms. However, the study also has some limitations. The sample was limited to two teaching hospitals affiliated with a single university, which may restrict the generalizability of the findings to other hospitals or healthcare systems. Additionally, the clinical capability assessment relied on self-reported measures rather than direct observational or objective data, which may not fully reflect actual practice and could overestimate participants’ behavior. Furthermore, sequential recruitment of the control and intervention groups may introduce temporal bias, as external conditions could have differed between the two recruitment periods. Finally, while the two-month follow-up provides short-term evidence of retention, longer-term follow-up would be necessary to determine the durability of the observed improvements over time.

## Conclusion

This study demonstrated that multimedia-based education significantly improved the clinical capability of operating room nurses in radiation protection, across multiple domains including knowledge, attitude, performance, and personal commitment. The positive effects of the intervention were evident not only immediately after the training but also at two-month follow-up, indicating short-term retention of learning. This educational approach appears to offer promising possibilities in facilitating access to radiation safety training, particularly in high-pressure clinical environments. Thus, integrating multimedia-based modules into continuing education programs is recommended to enhance the preparedness and safety practices of nurses involved in radiation-exposed procedures. Given the observed effectiveness of this approach in improving clinical capability, future research should examine long-term retention, as well as the integration of multimedia training with other pedagogical strategies to further support clinical performance and patient safety.

## Data Availability

The datasets are available from the corresponding author on reasonable request. De-identified data may be deposited in a public repository in the future, while ensuring participant confidentiality.

## Abbreviations

SPSS: Statistical Package for the Social Sciences
WHO: World Health Organization
ICRP: International Atomic Energy Agency
IAEA: International Commission on Radiological Protection
CI: Confidence Intervals
